# Digital Behavioral Risk Stratification Tool Integrated With HIV Online Self-Testing for HIV Positivity Among Men Who Have Sex With Men: Retrospective Observational Study

**DOI:** 10.2196/104883

**Published:** 2026-11-11

**Authors:** Wencheng Liu, Yan Jiang, Guanbin Zhang, Yi Lv

**Affiliations:** 1 National Center for AIDS/STD Control and Prevention, Chinese Center for Disease Control and Prevention, 155 Changbai Road, Changping District, Beijing, 102206, China, 86 18001131913; 2 National Key Laboratory of Intelligent Tracking and Forecasting for Infectious Diseases, Beijing, China; 3 Chinese Association of STD and AIDS Prevention and Control, Beijing, China; 4 School of Intelligent Medicine, Chengdu University of Traditional Chinese Medicine, Chengdu, China

**Keywords:** HIV self-testing, men who have sex with men, digital health, risk stratification, public health interventions

## Abstract

**Background:**

Due to concerns about privacy breaches and the fear of social stigma, some individuals engaging in high-risk behaviors—particularly men who have sex with men (MSM)—may avoid in-person HIV testing services and instead choose to purchase HIV self-test kits online. Unlike traditional e-commerce platforms that provide self-test kits directly without conducting risk assessments, the “Easy Test Know” platform integrates a structured behavioral risk assessment prior to purchasing an HIV self-test kit, allowing users to be categorized into different HIV risk groups.

**Objective:**

This study aimed to evaluate the performance of a digital HIV behavioral risk stratification tool integrated with online self-testing among MSM and to determine whether incorporating multidimensional behavioral indicators—particularly the high-risk venue diversity score—could improve the prediction of HIV positivity compared with the platform’s existing rule-based risk-scoring algorithm.

**Methods:**

This retrospective observational study used data from the “Easy Test Know” platform between November 2023 and February 2025. The analysis included 9961 MSM classified as medium or high risk who had a usable HIV test result. The continuous platform score and the deployed categorical output were evaluated, and enhanced and fully adjusted Firth logistic regression models were compared using areas under the receiver operating characteristic curves (AUCs) with stratified bootstrap 95% CIs, DeLong tests, calibration measures, and bootstrap optimism correction based on 1000 successful model refits.

**Results:**

Among 9961 participants, 9044 (90.8%) were classified as high risk, and 58 (0.58%) had an observed HIV-positive result. Positivity was 0.6% (n=54) in the high-risk group and 0.4% (4/914) in the medium-risk group, but the difference was not statistically significant (*P*=.70). The deployed categorical output had an AUC of 0.512 (95% CI 0.477‐0.539), with 93.1% (54/58) sensitivity and 9.2% (913/9903) specificity, whereas the continuous platform score had an AUC of 0.646 (95% CI 0.567‐0.725). Each 1-category increase in high-risk venue diversity was associated with higher odds of observed HIV positivity (odds ratio 2.04, 95% CI 1.53‐2.74; *P* for trend <.001). The enhanced and fully adjusted models had apparent AUCs of 0.728 (95% CI 0.656‐0.801) and 0.752 (95% CI 0.686‐0.817) and optimism-corrected AUCs of 0.688 and 0.713, respectively. Their optimism-corrected calibration slopes were 0.940 and 0.936.

**Conclusions:**

Compared with the platform’s original rule-based scoring system, the data-driven behavioral models incorporating high-risk venue diversity showed improved predictive performance, although bootstrap optimism correction yielded more conservative estimates. These findings support further development and independent validation of data-driven approaches to optimize digital HIV risk assessment tools.

## Introduction

Due to concerns about privacy breaches and social stigma, some high-risk individuals, especially men who have sex with men (MSM), may avoid offline HIV testing services and instead seek online HIV self-testing (HIVST) services [1-4]. Online HIVST offers a private and accessible complement to conventional testing and may reach individuals who are less likely to use in-person services [5-7]. Recent digitally supported and web-based HIVST programs have also demonstrated opportunities to connect self-testing with counseling, prevention, and linkage to care [8,9].

The “Easy Test Know” (Beijing Association for the Prevention and Control of Sexually Transmitted Diseases and AIDS) platform incorporates an HIV risk assessment module based on a structured behavioral questionnaire before users purchase HIVST kits, allowing users to be categorized into different HIV risk groups. This integrated approach combines digital behavioral assessment with online HIVST services and may provide a practical framework for targeted HIV prevention and testing interventions [6,10,11].

Previous studies on online HIVST have primarily examined acceptability, uptake, service delivery, or individual behavioral risk factors [8,9,12,13]. Existing HIV prediction studies have applied regression or machine learning approaches to behavioral data [14-16], but limited evidence is available for model development and internal validation within routine online HIVST services or for direct comparisons with deployed rule-based scores. Less is known about whether pretest digital risk scores correspond to observed HIV testing outcomes in routine service settings. Outcome-based evaluation is particularly important because expert-assigned weights and final risk categories may not preserve all the predictive information contained in the underlying behavioral data.

In addition, many existing HIV risk assessment approaches rely on isolated behavioral indicators and may not adequately capture cumulative exposure across multiple high-risk contexts. Individuals reporting exposure to multiple high-risk venues may engage in more complex behavioral patterns and broader social or sexual environments, potentially increasing HIV transmission risk [17-19]. Therefore, composite indicators reflecting multidimensional behavioral exposure may provide additional value for digital HIV risk prediction.

Using real-world data from the “Easy Test Know” platform, this study examined demographic and behavioral characteristics among MSM purchasing HIVST kits online. We further evaluated the association between the high-risk venue diversity score and HIV positivity and assessed whether behavioral reconstruction models could improve HIV risk prediction compared with the platform’s original rule-based scoring algorithm. Compared with previous studies, this study makes several notable contributions: (1) it is among the first real-world studies to systematically evaluate the consistency between digital HIV risk stratification and actual HIV positivity outcomes among MSM purchasing HIVST kits online; (2) it provides novel evidence on the association between the high-risk venue diversity score and HIV infection risk, including its dose-response relationship; and (3) it offers a comparative evaluation between the platform’s original rule-based composite scoring system and a data-driven behavioral modeling strategy constructed from the original behavioral variables, providing practical evidence for the optimization of digital HIV risk stratification tools.

Accordingly, the study evaluated the fixed platform score and deployed risk categories and developed 2 outcome-driven behavioral prediction models with bootstrap internal validation; no external validation data set was used.

## Methods

### Study Design and Data Source

This retrospective observational prediction model development and internal validation study used real-world data from the “Easy Test Know” online HIVST platform. Demographic, transaction, and behavioral data were collected from November 2023 to February 2025. The study was conducted through a single digital service platform serving users across China; because recruitment and data collection occurred online, no physical recruitment centers were involved. The platform integrates a structured HIV risk assessment questionnaire (Multimedia Appendix 1). Based on questionnaire responses, users were classified into low-, medium-, or high-risk categories.

### Recruitment and Data Collection

Recruitment occurred through the platform’s routine service pathway rather than a study-specific campaign. Users voluntarily accessed the service, and no additional participants were recruited, contacted, or assigned to an exposure by the research team. Records generated during routine use were screened against prespecified eligibility criteria for this retrospective secondary analysis.

### Study Population

The study population consisted of MSM who completed the platform-based HIV risk assessment and purchased HIVST kits. Participants were included if they were classified as medium or high risk, completed the HIVST pathway, and had an available, usable HIV test result recorded in the platform database. Purchasers without an available or usable test result were excluded rather than being classified as HIV-negative. Low-risk users generally did not proceed to kit purchase and therefore lacked an observed testing outcome within the platform; consequently, they could not be included in outcome-based predictive analyses. MSM eligibility was based on reported male-to-male sexual behavior rather than self-identified sexual orientation.

### Platform-Based HIV Risk Scoring Algorithm

The platform employs a rule-based HIV risk scoring algorithm (Multimedia Appendix 2) that assigns weighted scores based on demographic characteristics and HIV-related behavioral factors. Users are subsequently categorized into low-, medium-, or high-risk groups according to their final scores. The deployed platform calculated the dimension 1 and 2 base scores and applied its configured dimension 3 combination rules before storing the final continuous score and categorical output. The present study used these stored final outputs without modifying or recalculating the platform’s algorithm. The reconstructed Firth regression models were developed independently of the individual behavioral variables and did not reapply the platform’s dimension 3 rules.

### Firth Logistic Regression

The study used Firth logistic regression to address potential biases that may arise in standard logistic regression. Because HIV-positive outcomes were relatively rare in the study population, Firth logistic regression was used to reduce rare-event bias in parameter estimation and to provide finite estimates under complete or quasi-complete separation. This was relevant because only 58 HIV-positive outcomes were observed, and no HIV-positive outcomes occurred among participants reporting pre-exposure prophylaxis (PrEP) use. The fully adjusted model contained 24 predictor parameters (events-per-variable ratio, 2.42). Firth penalization was therefore used to support finite, less biased coefficient estimation in this rare-event setting.

### Behavioral Variables and High-Risk Exposure

The platform-based questionnaire collected information on HIV-related demographic and behavioral factors, including sexual orientation, condom use, HIV-positive partner status, number of sexual partners, commercial sex, history of sexually transmitted infections, use of new psychoactive drugs, sexual role, and group sex. The analytical dataset also contained recorded use of PrEP and postexposure prophylaxis (PEP), which were analyzed as separate variables. PEP and PrEP were recorded separately and were not components of the platform’s original scoring algorithm.

Sexual orientation was analyzed as a demographic characteristic distinct from the behavioral definition of MSM. The drug-use item asked whether participants had used new psychoactive drugs but did not capture specific substances, frequency, dose, or recency. The original partner-number response options were 2 to 5, 6 to 9, and greater than 10; the questionnaire did not provide categories for zero or one partner and left exactly 10 partners unspecified.

High-risk exposure was assessed based on 6 predefined location categories, including mobile social networking applications, online dating platforms, parks, bathhouses, public toilets, and bars/clubs. Because participants could report exposure to multiple venues, we constructed a composite indicator termed the high-risk venue diversity score, defined as the number of different high-risk venues reported by each participant (range: 0‐6). This indicator was intended to reflect cumulative exposure across multiple high-risk contexts [19,20]. “Only regular sexual partners” represented zero exposure and was not counted as an additional venue. Although “sex work venues” appeared in the questionnaire, no participant selected this option in the analytical dataset. This derived measure was distinct from the platform’s original weighted scoring algorithm.

### Outcome Measures

The primary outcome was HIV-positive status, defined as a participant-reported positive HIVST result submitted through the platform or a positive result from offline HIV testing reported after completion of the platform-based risk assessment. The outcome was taken directly from the recorded participant report, and no separate blinded outcome adjudication was performed. Participants without a usable returned result were excluded from the analytical dataset; therefore, the observed positivity proportion should not be interpreted as HIV prevalence among all platform purchasers or the broader MSM population.

### Study Size and Missing Data

The study size was determined pragmatically by all eligible records available in the supplied routine-service extract during the prespecified study period; no a priori sample size calculation was performed. Of 10,058 records screened, 87 records from female users were excluded, leaving 9971 male users meeting the platform-based MSM definition. Ten users without a usable self-reported HIVST or offline HIV test result were then excluded, resulting in the analytical sample of 9961; the usable-result return rate in this MSM pathway was therefore 99.9% (9961/9971), and 0.1% (10/9971) lacked a usable result. There were no missing values for the outcome or candidate model predictors in the analytical dataset; all model analyses used the same complete dataset, and no imputation was performed.

### Statistical Analysis

Categorical variables are presented as counts and percentages. Comparisons between medium- and high-risk groups and between the observed HIV-negative and HIV-positive groups were performed using chi-square or Fisher exact tests, as appropriate. The complete high-risk venue diversity score (0-6) was additionally evaluated for linear trend using Firth logistic regression, with the score treated as an ordinal continuous variable.

To evaluate HIV risk prediction performance, we constructed and compared three model specifications: (1) a baseline model that contained only the continuous platform risk scores; (2) an enhanced behavioral model that additionally incorporated the high-risk venue diversity scores; and (3) a fully adjusted behavioral model that incorporated additional HIV-related behavioral variables.

Because several behavioral variables had already been incorporated into the platform’s original scoring algorithm, the reconstructed models primarily evaluated whether data-driven weighting of behavioral information could improve predictive performance rather than the addition of entirely new predictors. The platform’s deployed categorical risk output was evaluated separately as an operational comparator.

The continuous platform score was modeled as a linear term. Age and all questionnaire-derived predictors were modeled using the predefined categories described in the *Result* section and Multimedia Appendix 2. In the reconstructed models, high-risk venue diversity was entered as 0, 1, or ≥2 venues; in a separate trend analysis, the complete score (0-6) was entered as an ordinal continuous variable. The same 9961 complete records were used in all model analyses.

Model discrimination was evaluated using the area under the receiver operating characteristic curve (AUC), and DeLong tests were used to compare AUCs between models. Stratified bootstrap resampling (2000 replicates) was used to estimate 95% CIs for apparent AUCs. Internal validation of the enhanced and fully adjusted Firth models used stratified bootstrap optimism correction. In each resample, the complete model was refitted in the bootstrap sample and evaluated in both the bootstrap sample and the original data; mean optimism was calculated as bootstrap-sample performance minus original-sample performance and subtracted from the apparent estimate. Sampling continued until 1000 successful refits were obtained for each model, and unsuccessful attempts were recorded. Optimism correction was applied to the AUC, calibration-in-the-large, calibration slope, expected-to-observed (E/O) ratio on the log scale, and Brier score. Hosmer-Lemeshow statistics were reported only for apparent calibration. Calibration plots display apparent and bootstrap bias-corrected curves based on 1000 resamples. The number of outcome events per estimated predictor parameter was calculated for each reconstructed model. All analyses were conducted using R software (version 4.5.2; R Foundation for Statistical Computing), and a 2-sided *P* value <.05 was considered statistically significant.

### Ethical Considerations

This study was approved by the Ethics Review Committee of the National Center for AIDS/Sexually Transmitted Disease Control and Prevention, Chinese Center for Disease Control and Prevention (approval number X131022302). The analytical dataset was deidentified before analysis and was accessible only to authorized study personnel; privacy and confidentiality were maintained throughout data handling and analysis. Participants received no additional compensation specifically for this retrospective secondary analysis. All study procedures were conducted in accordance with relevant guidelines and regulations.

## Results

### Analysis of Participant Characteristics and HIV Positivity

Of the 10,058 records in the supplied routine-service extract, 87 records from female users and 10 records from male users without a usable HIV test result were excluded. Among 9971 male users meeting the platform-based MSM definition, 9961 (99.9%) had a usable returned result. The final analysis included these 9961 MSM (Table 1), including 917 (9.2%) classified as medium risk and 9044 (90.8%) classified as high risk by the platform algorithm. Of these participants, 58 had an observed HIV-positive result and 9903 had an observed HIV-negative result.

**Table 1.** Participant characteristics by platform-assigned risk category among 9961 medium- and high-risk men who have sex with men (MSM), Easy Test Know online HIV self-testing platform, China, November 2023 to February 2025.

Variables	Medium risk (n=917)	High risk (n=9044)	*P* value
Age (y), n (%)			<.001^a^
<20	8 (0.9)	226 (2.5)	
20	76 (8.3)	1755 (19.4)	
30	671 (73.2)	6064 (67.0)	
40	135 (14.7)	808 (8.9)	
50	10 (1.1)	108 (1.2)	
60	17 (1.9)	83 (0.9)	
Sexual orientation, n (%)			.83
Uncertain	0 (0)	1 (0.0)	
Bisexual	10 (1.1)	83 (0.9)	
Homosexual	907 (98.9)	8960 (99.1)	
Sexual role, n (%)			<.001
Both	55 (5.9)	1189 (13.1)	
Receptive	96 (10.5)	4334 (47.9)	
Insertive	766 (83.5)	3521 (38.9)	
High-risk venue category^b^, n (%)			<.001
0	2 (0.2)	1 (0.0)	
1	614 (66.9)	2134 (23.6)	
≥2	301 (32.8)	6909 (76.4)	
Condom use, n (%)			<.001
Consistent use	14 (1.5)	12 (0.1)	
Occasional use	896 (97.7)	7631 (84.4)	
No use	7 (0.8)	1401 (15.5)	
HIV-positive partner, n (%)			<.001
Uncertain	836 (91.2)	8779 (97.1)	
No	81 (8.8)	264 (2.9)	
Yes	0 (0)	1 (0.0)	
Number of partners, n (%)			<.001
>10	0 (0)	8 (0.1)	
2‐5	911 (99.3)	7818 (86.4)	
6‐9	6 (0.7)	1218 (13.5)	
Commercial sex, n (%)			.01
No	917 (100)	8974 (99.2)	
Yes	0 (0)	70 (0.8)	
Group sex, n (%)			<.001
Uncertain	3 (0.3)	197 (2.2)	
No	887 (96.7)	8254 (91.3)	
Occasional	27 (2.9)	593 (6.6)	
STI^c^ history, n (%)			.65
No	917 (100)	9034 (99.9)	
Yes	0 (0)	10 (0.1)	
New psychoactive drug use, n (%)			<.001
No	509 (55.5)	841 (9.3)	
Yes	408 (44.5)	8203 (90.7)	
PEP^d^ use, n (%)			.001
No	915 (99.8)	9044 (100)	
Yes	2 (0.2)	0 (0)	
PrEP^e^ use, n (%)			.70
No	836 (91.2)	8205 (90.7)	
Yes	81 (8.8)	839 (9.3)	
HIV-positive, n (%)			.70
No	913 (99.6)	8990 (99.4)	
Yes	4 (0.4)	54 (0.6)	

a *P* values were calculated using chi-square tests or Fisher exact tests, as appropriate.

b High-risk venue diversity score was categorized based on the number of reported high-risk venue types.

c STI: sexually transmitted infection.

d PEP: postexposure prophylaxis.

e PrEP: pre-exposure prophylaxis.

Compared with participants in the medium-risk group, those classified as high risk differed across multiple behavioral characteristics, including sexual role, high-risk venue diversity, condom use, HIV-positive partner status, number of sexual partners, commercial sex, group sex, drug use, and PEP use (Table 1). These differences describe how the rule-based platform score stratified behavioral profiles.

The distribution of high-risk venue exposure differed substantially between groups. Participants in the high-risk group were considerably more likely to report exposure to 2 or more high-risk venues compared with those in the medium-risk group (6909/9044, 76.4% vs 301/917, 32.8%; *P*<.001), indicating substantial differences in cumulative behavioral exposure patterns between the 2 risk categories.

Among the 9961 participants, 58 had an observed positive HIV test result, corresponding to an observed positivity proportion of 0.58% (Table 2). Participants classified as high risk had a numerically higher positivity proportion than those classified as medium risk (54/9044, 0.6% vs 4/917, 0.4%), but the difference was not statistically significant. The observed positivity proportion reflects only participants with available test results and should not be interpreted as HIV prevalence among all platform users or the broader MSM population.

**Table 2.** Participant characteristics by observed HIV test result among 9961 medium- and high-risk men who have sex with men (MSM), Easy Test Know online HIV self-testing platform, China, November 2023 to February 2025.

Variables	Negative (n=9903)	Positive (n=58)	*P* value
Age (y), n (%)			.048
<20	234 (2.4)	0 (0)	
20	1815 (18.3)	16 (27.6)	
30	6704 (67.7)	31 (53.4)	
40	934 (9.4)	9 (15.5)	
50	116 (1.2)	2 (3.4)	
60	100 (1.0)	0 (0)	
Sexual orientation, n (%)			.76
Uncertain	1 (0.0)	0 (0)	
Bisexual	93 (0.9)	0 (0)	
Homosexual	9809 (99.1)	58 (100)	
Sexual role, n (%)			.39
Both	1238 (12.5)	6 (10.3)	
Receptive	4399 (44.4)	31 (53.4)	
Insertive	4266 (43.1)	21 (36.2)	
Platform risk level, n (%)			.70
Medium risk	913 (9.2)	4 (6.9)	
High risk	8990 (90.8)	54 (93.1)	
High-risk venue category, n (%)			.01
0	3 (0.0)	0 (0)	
1	2742 (27.7)	6 (10.3)	
≥2	7158 (72.3)	52 (89.7)	
Condom use, n (%)			.33
Consistent use	26 (0.3)	0 (0)	
Occasional use	8481 (85.6)	46 (79.3)	
No use	1396 (14.1)	12 (20.7)	
HIV-positive partner, n (%)			<.001
Uncertain	9571 (96.6)	44 (75.9)	
No	332 (3.4)	13 (22.4)	
Yes	0 (0)	1 (1.7)	
Number of partners, n (%)			<.001
>10	4 (0.0)	4 (6.9)	
2‐5	8688 (87.7)	41 (70.7)	
6‐9	1211 (12.2)	13 (22.4)	
Commercial sex (%)			.001
No	9836 (99.3)	55 (94.8)	
Yes	67 (0.7)	3 (5.2)	
Group sex, n (%)			.008
Uncertain	200 (2.0)	0 (0.0)	
No	9092 (91.8)	49 (84.5)	
Occasional	611 (6.2)	9 (15.5)	
STI^a^ history, n (%)			<.001
No	9897 (99.9)	54 (93.1)	
Yes	6 (0.1)	4 (6.9)	
New psychoactive drug use, n (%)			>.99
No	1342 (13.6)	8 (13.8)	
Yes	8561 (86.4)	50 (86.2)	
PEP^b^ use, n (%)			>.99
No	9901 (99.9)	58 (100)	
Yes	2 (0.0)	0 (0)	
PrEP^c^ use, n (%)			.03
No	8983 (90.7)	58 (100)	
Yes	920 (9.3)	0 (0)	

a STI: sexually transmitted infection.

b PEP: postexposure prophylaxis.

c PrEP: pre-exposure prophylaxis.

The continuous platform risk score showed modest discrimination for observed HIV positivity (AUC 0.646, 95% CI 0.567‐0.725; Table S1 in Multimedia Appendix 3). When the deployed categorical risk output was evaluated directly, its AUC was 0.512 (95% CI 0.477‐0.539). The categorical output achieved high sensitivity (93.1%), classifying 54 of 58 HIV-positive participants as high risk, although its specificity was 9.2% (913/9903); the remaining 4 HIV-positive participants were classified as medium risk.

Observed HIV positivity increased across the well-populated levels of the high-risk venue diversity score: 0% (0/3) at score 0, 0.2% (6/2748) at score 1, 0.5% (18/3824) at score 2, 0.8% (21/2762) at score 3, and 2.1% (13/623) at score 4; only one participant had a score of 5, and none had a score of 6 (Table S4 in Multimedia Appendix 3). In Firth logistic regression, each 1-category increase in the diversity score was associated with higher odds of observed HIV positivity (odds ratio [OR] 2.04, 95% CI 1.53‐2.74; *P* for trend <.001).

HIV-positive partner status was strongly associated with observed HIV positivity in the fully adjusted model (Table 3), although estimates were imprecise for the very small “yes” group. Compared with participants who were uncertain about their partner’s HIV status, the adjusted OR was 6.57 (95% CI 2.87‐13.58) for those reporting no HIV-positive partner and 172.22 (95% CI 7.76‐27,046.77) for those reporting an HIV-positive partner. PrEP use was inversely associated with observed HIV positivity: none of the 920 PrEP users had a positive result, compared with 58 positive results among 9041 nonusers (adjusted OR 0.10, 95% CI 0.001‐0.68; *P*=.01). This complete separation was one rationale for the use of Firth regression and should not be interpreted as a causal estimate of PrEP effectiveness.

**Table 3.** Fully adjusted Firth logistic regression model for observed HIV positivity among 9961 medium- and high-risk men who have sex with men (MSM), Easy Test Know online HIV self-testing platform, China, November 2023 to February 2025.

Variables	β coefficient^a^	OR^b^ (95% CI**)**	*P* value
Intercept			
Constant	1.821960	—^c^	—
Age (y)			
<20	0.000000	Reference^d^	—
20‐29	1.359521	3.89 (0.52‐498.88)	.24
30‐39	0.759321	2.14 (0.30‐271.54)	.55
40‐49	1.410274	4.10 (0.50‐531.29)	.23
50‐59	0.422914	1.53 (0.004‐320.85)	.85
≥60	0.809970	2.25 (0.01‐417.79)	.69
Sexual orientation			
Uncertain	0.000000	Reference	—
Bisexual	–5.441295	0.004 (0.000‐1.38)	.06
Gay	–4.581704	0.01 (0.000‐1.82)	.07
High-risk venue category			
0 types	0.000000	Reference	—
1 type	–3.907189	0.02 (0.001‐5.29)	.13
≥2 types	–2.969411	0.05 (0.002‐13.01)	.23
Condom use			
Consistent use	0.000000	Reference	—
Occasional use	3.677832	39.56 (1.18‐14,271.82)	.04
No use	4.218966	67.96 (1.88‐25,170.82)	.02
HIV-positive partner			
Uncertain	0.000000	Reference	—
No	1.881925	6.57 (2.87‐13.58)	<.001
Yes	5.148746	172.22 (7.76‐27,046.77)	.002
Number of partners			
>10	0.000000	Reference	—
2‐5	–4.740278	0.009 (0.002‐0.05)	<.001
6‐9	–4.334894	0.01 (0.002‐0.08)	<.001
Commercial sex			
No	0.000000	Reference	—
Yes	–0.289085	0.75 (0.11‐3.21)	.72
STI^e^ history			
No	0.000000	Reference	—
Yes	4.641172	103.67 (7.77‐14,296.85)	<.001
New psychoactive drug use			
No	0.000000	Reference	—
Yes	0.144633	1.16 (0.55‐2.79)	.72
Sexual role			
Both insertive and receptive	0.000000	Reference	—
Receptive	0.081073	1.08 (0.48‐2.81)	.85
Insertive	–0.269181	0.76 (0.33‐2.03)	.57
Group sex			
Uncertain	0.000000	Reference	—
No	0.559976	1.75 (0.25‐222.00)	.67
Occasional	1.135768	3.11 (0.35‐410.44)	.37
PEP^f^ use			
No	0.000000	Reference	—
Yes	4.579115	97.43 (0.45‐21,356.33)	.08
PrEP^g^ use			
No	0.000000	Reference	—
Yes	–2.328992	0.10 (0.001‐0.68)	.01

a β coefficients are on the log-odds scale.

b OR: odds ratio.

c Not applicable.

d Categorical predictors use indicator coding, with the category labeled “Reference” as the reference level.

e STI: sexually transmitted infection.

f PEP: postexposure prophylaxis.

g PrEP: pre-exposure prophylaxis.

### Model Comparison and Optimization

#### Model Discriminatory Ability

The enhanced behavioral model achieved an apparent AUC of 0.728 (95% CI 0.656‐0.801; Table S1 in Multimedia Appendix 3), compared with 0.646 (95% CI 0.567‐0.725) for the continuous platform score, corresponding to a ΔAUC of +0.082. The DeLong test indicated significantly better apparent discrimination for the enhanced model (*P*=.01; Table S2 in Multimedia Appendix 3).

The fully adjusted Firth model achieved the highest apparent AUC of 0.752 (95% CI 0.686‐0.817; Table S1 in Multimedia Appendix 3) and also significantly outperformed the continuous platform score by DeLong test (*P*<.001; Table S2 in Multimedia Appendix 3).

Bootstrap internal validation identified mean AUC optimism of 0.040 for the enhanced behavioral model and 0.039 for the fully adjusted Firth model, yielding optimism-corrected AUCs of 0.688 and 0.713, respectively (Table S1 in Multimedia Appendix 3). The continuous platform score and deployed categorical output were fixed platform measures rather than models estimated in this dataset and therefore were not optimism-corrected.

The fitted intercepts were 1.972304 for the enhanced model and 1.821960 for the fully adjusted model. Table 3 and Table S5 in Multimedia Appendix 3 provide the exact log-odds coefficients and complete specifications for the fully adjusted and enhanced behavioral models, respectively. To calculate an individual predicted probability, code each categorical predictor using the reference category listed in the corresponding table, calculate the linear predictor, η = β_0_ + Σβ_j_X_j_, and transform it as *P* = exp(η)/[1 + exp(η)]. These equations describe model use for prediction; they do not establish a clinical decision threshold.

Receiver operating characteristic analyses (Figure 1) showed that the reconstructed behavioral models improved discrimination compared with the continuous platform score, whereas the deployed categorical output emphasized sensitivity (54/58, 93.1%) over specificity (913/9903, 9.2%). The continuous platform score was retained as the primary baseline for model comparison because it preserves the information used by the platform before categorization.

**Figure 1.**
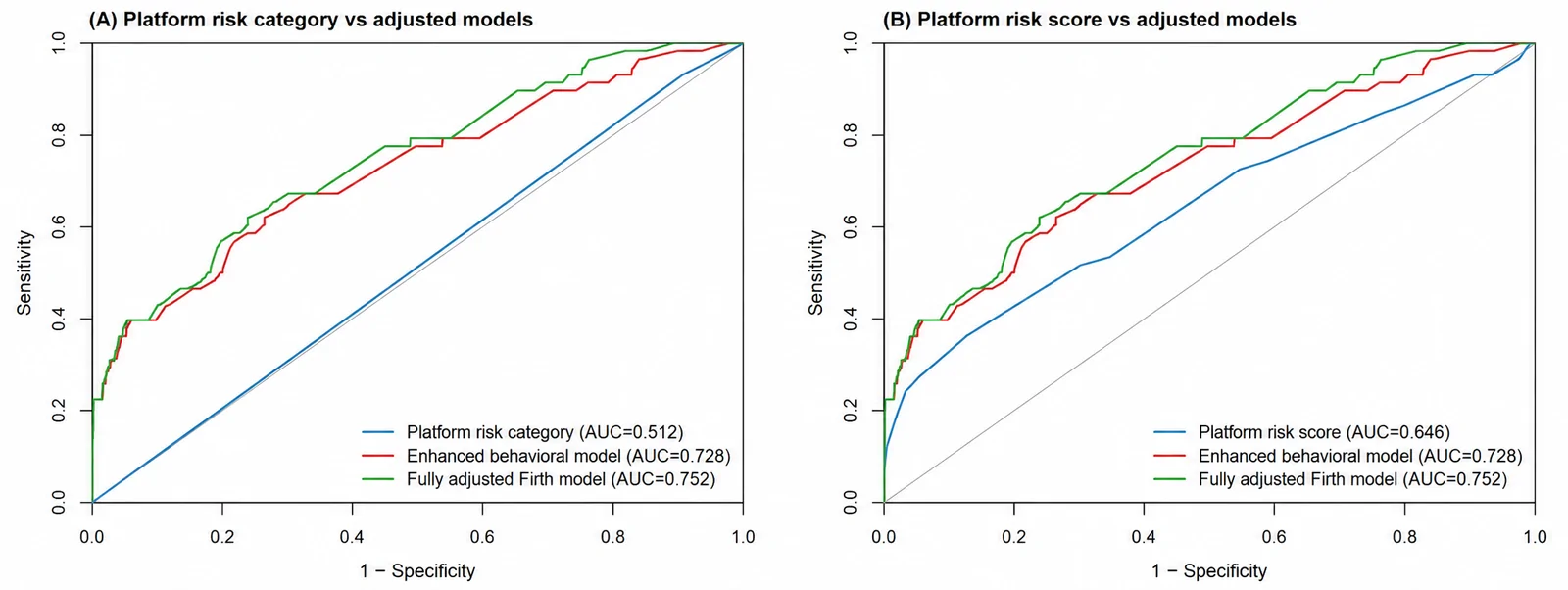
Receiver operating characteristic curves for adjusted models vs platform model among 9961 medium- and high-risk men who have sex with men, Easy Test Know online HIV self-testing platform, China, November 2023 to February 2025. AUC: area under the receiver operating characteristic curve.

#### Model Calibration Performance

Both reconstructed models showed favorable apparent calibration in the development data (Table S3 in Multimedia Appendix 3). The enhanced behavioral model had a calibration-in-the-large of –0.149, a calibration slope of 1.031, an E/O ratio of 1.144, a Brier score of 0.005102, and a Hosmer-Lemeshow *χ*^2^_7_=7.007 (*P*=.43). Corresponding values for the fully adjusted Firth model were –0.162, 1.035, 1.158, 0.005103, and *χ*^2^_8_=7.121 (*P*=.52).

After bootstrap optimism correction, the enhanced model had a calibration-in-the-large of –0.141, a calibration slope of 0.940, an E/O ratio of 1.137, and a Brier score of 0.005273. Corresponding corrected values for the fully adjusted model were –0.152, 0.936, 1.149, and 0.005292 (Table S3 in Multimedia Appendix 3). Together, these findings suggest that the reconstructed models largely retained favorable calibration after adjustment for optimism. The calibration plots additionally display apparent and bootstrap bias-corrected curves based on 1000 resamples (Figure 2).

**Figure 2.**
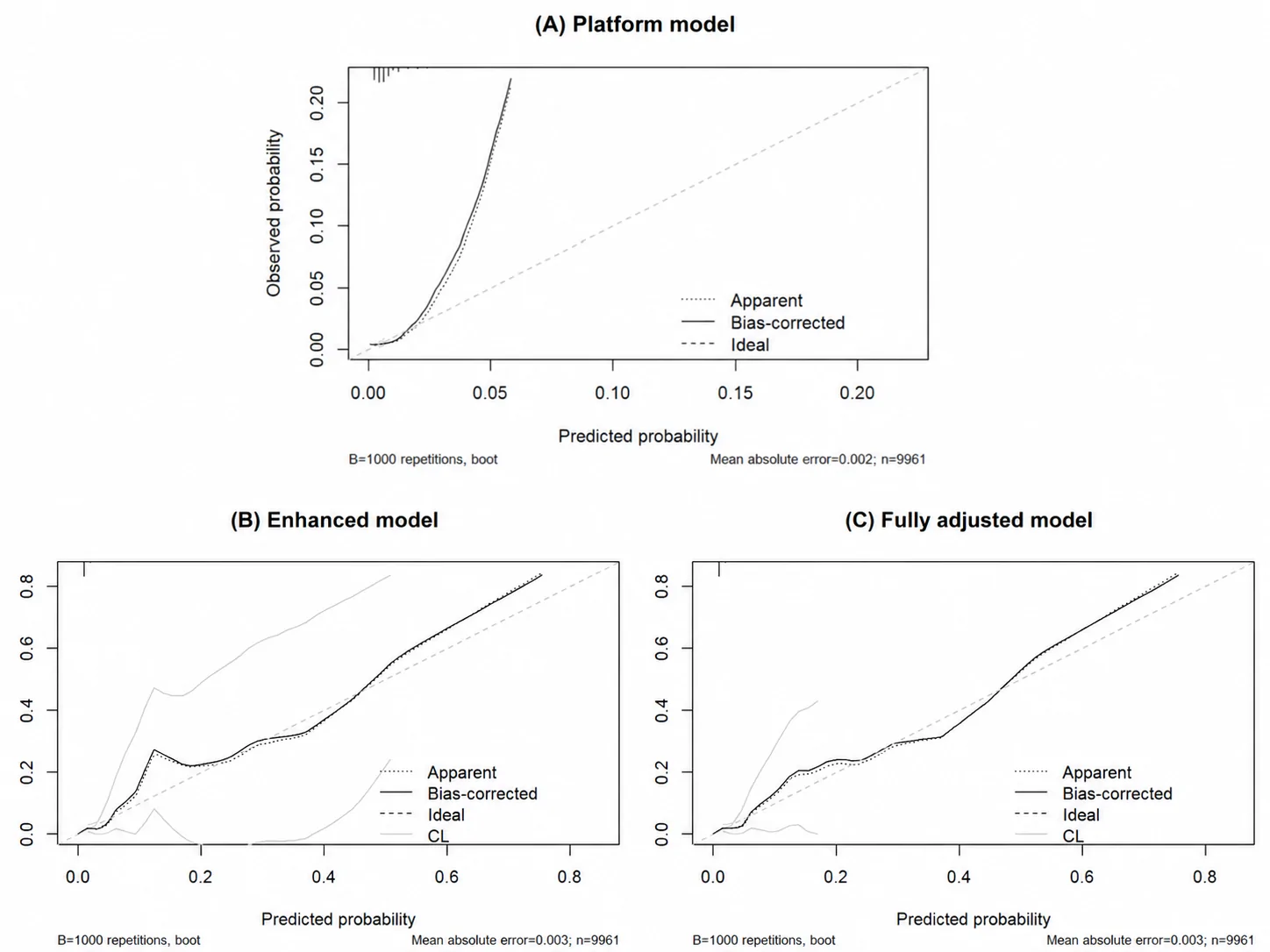
Calibration curves for adjusted models vs platform model among 9961 medium- and high-risk men who have sex with men, Easy Test Know online HIV self-testing platform, China, November 2023 to February 2025. CL: confidence limits.

The enhanced behavioral model included 22 predictor parameters for 58 HIV-positive events (2.64 events per parameter), and the fully adjusted model included 24 predictor parameters (2.42 events per parameter). Because several predictor categories were sparsely populated, obtaining 1000 successful bootstrap refits required 2623 attempts for the enhanced model and 2944 attempts for the fully adjusted model. The prespecified target of 1000 successful refits was achieved for both models. Although optimism correction appropriately produced more conservative estimates, the fully adjusted model retained the highest AUC, and both reconstructed models maintained calibration slopes close to 1. Thus, the internally validated results preserved the overall performance advantage of the reconstructed models, although external validation in larger datasets is needed to confirm and further refine these estimates.

## Discussion

### Principal Findings

This study evaluated the performance of a digital HIV behavioral risk stratification system integrated with online HIVST services among MSM with available test results in the medium- and high-risk pathways. The deployed categorical output showed high sensitivity but low specificity, whereas the continuous platform score provided modest discrimination. Outcome-driven behavioral models achieved higher apparent AUCs and retained improved AUCs after bootstrap optimism correction, and high-risk venue diversity was associated with observed HIV positivity. The relatively low observed positivity proportion (58/9961, 0.58%) may partly reflect the platform’s broad, prevention-oriented case-finding strategy, which prioritized sensitivity (54/58, 93.1%) over specificity (913/9903, 9.2%) and therefore included many HIV-negative users rather than restricting testing to a narrowly selected clinical population. These findings address the study aims but apply only to the analyzed population and should not be interpreted as population prevalence or performance across the full risk spectrum.

The categorical and continuous platform results require separate interpretation. Positivity was 0.6% (54/58) in the high-risk group and 0.4% (4/914) in the medium-risk group, but the difference was not statistically significant. This nonsignificant difference between categories does not establish that the platform lacks practical value; rather, it supports evaluating and refining its thresholds using observed outcome data.

The observed association between exposure to multiple high-risk venues and HIV positivity suggests that cumulative venue-based behavioral exposure may provide additional information beyond conventional individual behavioral indicators. These findings support the potential value of integrating multidimensional behavioral exposure measures into digital HIV risk assessment frameworks.

### Potential Mechanism for the High-Risk Venue Diversity Score

The association between high-risk venue diversity score and HIV positivity may reflect broader patterns of cumulative behavioral exposure. On the one hand, different types of high-risk sites may offer greater anonymity, more frequent temporary partner contacts, and environments where continuous risk prevention is difficult to maintain. Exposure to multiple sites often places individuals in more complex and overlapping high-risk sexual networks. On the other hand, the number of different sites individuals visit may reflect several risk dimensions, such as the frequency of behaviors, sexual behavior diversity, and the degree of social network overlap. Compared with a single behavioral indicator, this metric provides a more comprehensive reflection of cumulative risk intensity, which may help explain the dose-response relationship for HIV positivity [18,20,21]. Because these mechanisms were not measured directly, the venue diversity finding should be interpreted as predictive rather than causal. Nevertheless, it suggests that venue-based exposure measures may contribute additional information to digital HIV risk assessment beyond conventional single-behavior indicators [14,15].

### Interpretation of Drug Use Findings

Although drug use was more common among participants classified as high risk by the platform algorithm, it was not significantly associated with observed HIV positivity. The questionnaire item combined new psychoactive drugs but did not capture specific substances, frequency, dose, or recency. Accordingly, the high reported proportion should not be generalized to the broader MSM population, and the null association may reflect limited measurement specificity or statistical power rather than the absence of a relationship with HIV risk.

One possible explanation is that drug use may reflect the clustering of multiple co-occurring high-risk behaviors within the platform’s scoring framework. However, after adjustment for other behavioral factors, its additional predictive contribution appeared limited. These findings suggest that the relative importance of specific behavioral indicators may differ between expert-defined scoring systems and data-driven predictive models.

### Comparison With Existing Studies

Compared with many previous studies focusing on individual behavioral factors or offline testing populations [22], the present study evaluated behavioral risk prediction within a real-world online HIVST platform. The observed association between exposure to multiple high-risk venues and HIV positivity is broadly consistent with previous studies suggesting that repeated exposure across diverse sexual or social environments may be associated with increased HIV risk.

In addition, the use of real-world platform-based behavioral and transaction data provides insight into HIVST users who may be less engaged with conventional in-person testing services. Recent digitally supported HIVST programs have also shown that online self-testing can be connected to counseling, prevention, and linkage to care [8,9]. The present study complements this literature by evaluating the correspondence between a prepurchase behavioral assessment and available testing outcomes.

### Implications of Digital Risk Stratification

Importantly, the improved performance observed in the reconstructed behavioral models should not be interpreted simply as the addition of entirely new predictors. Several behavioral variables included in the optimized models were already incorporated into the platform’s original rule-based scoring system. Rather, the observed improvement likely reflects differences in how behavioral information was aggregated and weighted. The platform algorithm relies on predefined, expert-assigned weights, whereas the present study applied a data-driven regression framework to estimate the relative contributions of behavioral variables directly from observed outcome data. These findings suggest that optimizing weighting strategies and behavioral feature integration may substantially improve the predictive performance of digital HIV risk stratification tools. It should be noted that the comparison is between a model optimized on the current dataset and a fixed algorithm; the observed improvement partly reflects the statistical advantage of in-sample optimization and does not imply that the platform algorithm is deficient.

From an application standpoint, the reconstructed behavioral models demonstrated improved discrimination compared with the continuous platform score, and the fully adjusted model retained the highest AUC after bootstrap optimism correction (0.713). The optimism-corrected calibration slopes also remained close to 1, supporting the potential value of integrating multidimensional behavioral indicators and outcome-informed weighting into digital HIV risk stratification. These models may provide a useful foundation for refining risk assessment and identifying individuals who could benefit from prioritized follow-up testing, online counseling, self-test support, or linkage to PrEP and other prevention services, particularly in resource-limited settings [8,14,16,23,24]. In a national-level implementation study in Vietnam [9], an online HIVST distribution program successfully linked 97.5% of individuals with reactive results to treatment, while approximately 20% of those with negative results initiated or continued PrEP. This experience further illustrates how appropriately validated digital risk assessment tools could be integrated into the HIV detection, prevention, and treatment continuum. Further external validation of the present models would help confirm their performance and inform appropriate implementation thresholds.

### Limitations

This study has several limitations. First, outcome ascertainment depended on a usable self-test result returned through the platform or an offline result reported after assessment. Participants without usable returned results were excluded rather than classified as HIV-negative, but selective result reporting may still affect the observed positivity proportion and model coefficients. The findings should therefore not be interpreted as HIV prevalence among all purchasers or the broader MSM population.

Second, low-risk users were not represented because they generally did not proceed through the same purchase and result-return pathway. Restricting the analysis to medium- and high-risk participants compressed the evaluated score range; consequently, the reported platform performance applies only to the observed risk spectrum and not to all platform users.

Third, only 58 HIV-positive outcomes were available, and the fully adjusted model included 24 predictor parameters, corresponding to 2.42 events per parameter. Firth penalization reduced coefficient bias and provided finite estimates under separation, but it did not eliminate overfitting. Bootstrap internal validation yielded more conservative AUC estimates, while the corrected calibration slopes remained close to 1; however, the limited number of events and sparse predictor categories affected the stability of bootstrap refitting. Independent validation is therefore needed to confirm the observed performance gains.

Finally, the behavioral data were self-reported and may have been affected by recall or social desirability bias. Several questionnaire items were broad or sparsely populated, and the observational design does not support causal interpretation of the reported associations.

### Future Research Directions

Future research should externally validate the continuous score, deployed categories, and more parsimonious outcome-driven models in larger independent cohorts that include the full risk spectrum and more complete outcome ascertainment. Temporal or geographic validation and assessment of alternative thresholds would help determine whether the optimism-corrected performance gains translate into useful service decisions.

Longitudinal studies should also examine the timing and frequency of venue exposure and incorporate partner- or network-level information. As digital HIVST interventions enter the regulatory landscape, standardized approaches to model validation, updating, privacy, and fairness will also be important [25].

### Conclusions

Among MSM who entered the medium- and high-risk online HIVST pathway and had available test results, high-risk venue diversity was associated with observed HIV positivity, and outcome-driven behavioral models achieved higher apparent discrimination than the platform’s continuous rule-based score. After bootstrap optimism correction, the fully adjusted model retained the highest AUC (0.713), and the corrected calibration slopes for both reconstructed models remained close to 1, supporting the potential value of data-driven behavioral weighting for digital HIV risk stratification. The deployed categorical output retained high sensitivity, indicating value for broad risk capture, although its low specificity suggests that the operating threshold could be refined. Taken together, these findings support the feasibility of using routinely collected behavioral and testing outcomes to iteratively evaluate and update digital risk-stratification tools. With further validation, such tools may help online HIVST programs prioritize follow-up testing, tailored counseling, prevention support, and linkage to care, complementing digitally supported HIVST service models reported previously [8,9]. This study therefore provides a practical framework for optimizing rule-based digital assessment systems while preserving accessible self-testing pathways. Because low-risk users and purchasers without usable results were not represented and only 58 positive outcomes were observed, independent validation, broader outcome ascertainment, and prospective assessment of decision thresholds are needed before programmatic implementation. In practice, simply counting the number of distinct high-risk venues a user reports could serve as an easily collectible proxy to prioritize resource allocation for follow-up counseling.

## Supplementary material

10.2196/104883 Multimedia Appendix 1 HIV risk assessment questionnaire.

10.2196/104883 Multimedia Appendix 2 Quantitative algorithms and scoring systems for HIV risk assessment.

10.2196/104883 Multimedia Appendix 3 Model performance, HIV positivity by high-risk venue diversity, and Firth logistic regression results among men who have sex with men using the Easy Test Know online HIV self-testing platform in China, November 2023 to February 2025.

## Abbreviations

AUC: area under the receiver operating characteristic curve
E/O: expected-to-observed
HIVST: HIV self-testing
MSM: men who have sex with men
OR: odds ratio
PEP: postexposure prophylaxis
PrEP: pre-exposure prophylaxis
