# Supplementary material for: Digital Behavioral Risk Stratification Tool Integrated With HIV Online Self-Testing for HIV Positivity Among Men Who Have Sex With Men: Retrospective Observational Study
Source: J Med Internet Res. 2026 Nov 11;28:e104883. doi: 10.2196/104883 (PMC13754183; doi:10.2196/104883)
Supplement: Supplementary file 1 [file jmir-v28-e104883-s001.doc]

**HIV Risk Assessment Questionnaire**

**I. Basic Risk Variables**

1. Gender

☐ Male

☐ Female

2. Sexual Orientation

☐ Gay

☐ Straight

☐ Bisexual

☐ Unsure

3. Date of Birth (Please provide your date of birth)

______ Year / __ Month / __ Day

**II. Core Behavioral Risks**

4. How You Find Partners

☐ Mobile social networking applications

☐ Online dating platforms

☐ Parks

☐ Bathhouses

☐ Public toilets

☐ Bars

☐ Sex work venues

☐ Only regular sexual partners

5. Condom Use

☐ Always use

☐ Sometimes use

☐ Never use

6. HIV-Positive Partners

☐ None

☐ Unknown

☐ Yes

7. Number of Sexual Partners

☐ 2–5

☐ 6–9

☐ >10

8. Commercial Sex Work

☐ No

☐ Yes

9. History of STIs (Sexually Transmitted Infections)

☐ No

☐ Yes

10. Use of New Psychoactive Substances

☐ No

☐ Yes

11. Sexual Role

☐ Top

☐ Both Top and Bottom

☐ Bottom

12. Participation in Group Sex

☐ No

☐ Occasionally

☐ Uncertain
