# Supplementary material for: Digital Behavioral Risk Stratification Tool Integrated With HIV Online Self-Testing for HIV Positivity Among Men Who Have Sex With Men: Retrospective Observational Study
Source: J Med Internet Res. 2026 Nov 11;28:e104883. doi: 10.2196/104883 (PMC13754183; doi:10.2196/104883)
Supplement: Supplementary file 2 [file jmir-v28-e104883-s002.doc]

### Quantitative Algorithms and Scoring Systems for HIV Risk Assessment

**I. Core Premise: General Weighted Scoring Logic of the Platform**

#### The platform used a weighted scoring approach in which predefined points were assigned to questionnaire responses and the final score was mapped to a risk category. The following sections reproduce the platform algorithm and scoring rules.

#### II. The “Three-Dimensional Scoring System” for Risk Assessmen

**Dimension 1: Basic Risk Variables (2–5 points; affects the overall risk baseline)**

| Indicator | Option | Risk Score | Rationale |
| --- | --- | --- | --- |
| Gender + Sexual Orientation | Male + Gay / Bisexual (engages in same-sex sexual activity) | 3 points | Male-to-male sexual activity (anal intercourse) carries the highest physiological risk |
|  | Male + Heterosexual / Undetermined (no same-sex sexual activity) | 1 point | Heterosexual sexual behavior carries lower risk than male-to-male sexual behavior |
| Age (derived from date of birth) | 18–30 years old | 2 points | High sexual partner turnover and low awareness of protection in this age group |
|  | ≤17 years old or ≥31 years old | 0 points | Relatively higher sexual partner stability (not absolute) |

**Dimension 2: Core Behavioral Risks (1–15 points, core factors determining risk)**

| Question No. | Option Category | Risk Score |
| --- | --- | --- |
| 04 (Method of Finding Partners) | Only a steady sexual partner | 0 points |
|  | Traditional computer dating software | 2 points |
|  | Mobile social apps / Bars and clubs | 4 points |
|  | Parks / Bathhouses / Public Restrooms | 6 points |
|  | Sex work venues (e.g., brothels) | 8 points |
| 05 (Condom Use) | Always use | 0 points |
|  | Sometimes use | 3 points |
|  | Never use | 6 points |
| 06 (HIV-Positive Partner) | None | 0 points |
|  | Unknown | 2 points |
|  | Yes | 5 points |
| 07 (Number of Sexual Partners) | 2-5 | 1 point |
|  | 6-9 | 3 points |
|  | >10 | 5 points |
| 08 (Commercial Sex) | No | 0 points |
|  | Yes | 6 points |
| 09(Past STIs) | No | 0 points |
|  | Yes | 4 points |
| 10(New psychoactive substances) | No | 0 points |
|  | Yes | 3 points |
| 11 (Sexual roles, gay men) | Active Partner | 0 points |
|  | Active + Receptive Partner | 2 points |
|  | Receptive Partner | 4 points |
| 12 (Group Sex) | No | 0 points |
|  | Occasional | 2 points |
|  | Frequent | 4 points |

**Dimension 3: Key Risk Multipliers (Risk factor ×1.5–2; escalates to high-risk scenarios)**

If the following “key risk combinations” are met simultaneously, the **corresponding multiplier** must be applied to the total score (to reflect the “severity of cumulative risk”):

- Combination 1: HIV-positive partner + never used condoms → Total score × 2.0 (direct high-risk exposure)
- Combination 2: Receptive partner in MSM relationships + never used condoms → Total score × 1.8 (Biological risk + Unprotected sex)
- Combination 3: Exposure in commercial sex settings + History of STI infection → Total score × 1.5 (High-risk setting + Mucosal damage)
- Dimension 3 applies additional weighting to the score derived from Dimensions 1 and 2 according to predefined combinations of behavioral characteristics and is part of the platform's original risk-scoring algorithm.
- The study evaluated the stored final platform score and category, so any applicable Dimension 3 multiplier was already reflected in the platform output used in the analysis. The Firth logistic regression models were independently constructed using individual behavioral variables and did not reapply these multipliers.

### III. “Score Classification Criteria” for Low / Medium / High Risk (Final score after any applicable Dimension 3 multiplier)

| Risk Level | Final Score Range | Key Characteristics |
| --- | --- | --- |
| Low Risk | 0-5 points | Stable sexual partner, consistent condom use, no high-risk behaviors |
| Medium Risk | 6-15 points | Occasional unprotected sex, 6–9 sexual partners, contact with anonymous partners |
| High Risk | >15 points | Meets one or more “critical risk combinations,” or multiple high-risk behaviors combined |

### IV. Practical Examples: How to Calculate Risk Using the Algorithm

#### Example 1: Low-Risk Individual (Male, 25 years old, heterosexual)

- Dimension 1: Gender + Sexual Orientation (1 point) + Age (2 points) → 3 points
- Dimension 2: Only one steady sexual partner (0) + Always uses condoms (0) + Number of sexual partners < 5 (1) + No other high-risk behaviors → 1 point
- Final Score: 3 + 1 = 4 points → Low Risk

#### Example 2: High-risk group (Male, 28 years old, gay)

- Dimension 1: Gender + Sexual orientation (3 points) + Age (2 points) → 5 points
- Dimension 2: Seeking partners via mobile dating apps (4) + Sometimes uses condoms (3) + Number of sexual partners > 10 (5) + Receptive partner (4) + Occasional group sex (2) → 18 points
- Final score: 5+18=23 points → High risk

### V. Important Notes: “Boundaries and Limitations” of the Algorithm

1. This is a “simplified model”: Actual public health assessments incorporate more detailed indicators such as regional epidemic data (e.g., local HIV prevalence) and behavioral frequency (e.g., number of unprotected encounters per month);
2. Score ≠ Diagnosis: A high-risk score merely indicates a “higher probability of infection”; HIV testing (e.g., antibody testing, nucleic acid testing) is required to confirm infection;
3. Risk can be reduced through protection: Even with a high score, consistently practicing “consistent condom use + having a steady sexual partner” in the future can significantly reduce risk (equivalent to “dynamic score reduction”).

**Note on the source of the platform risk-assessment information**

Appendix 2 reproduces the original risk-assessment algorithm and explanatory information provided by the online HIV self-testing platform. The point values and categories are operational scoring rules rather than independently validated estimates of HIV transmission probability. The legacy platform documentation listed a “Frequent” option for group sex, whereas the analytical dataset contained “Uncertain,” “No,” and “Occasional”; no analytical category was collapsed.
