# Supplementary material for: Digital Behavioral Risk Stratification Tool Integrated With HIV Online Self-Testing for HIV Positivity Among Men Who Have Sex With Men: Retrospective Observational Study
Source: J Med Internet Res. 2026 Nov 11;28:e104883. doi: 10.2196/104883 (PMC13754183; doi:10.2196/104883)
Supplement: Supplementary file 3 [file jmir-v28-e104883-s003.doc]

**Table S1. Apparent and bootstrap optimism-corrected discrimination of platform risk models and reconstructed behavioral models among MSM, Easy Test Know online HIVST platform, China, November 2023-February 2025**

| **Model** | **Apparent AUC^[1]^** | **Bootstrap 95% CI^[2]^** | **Mean optimism** | **Optimism-corrected AUC** | **Successful refits / attempts** |
| --- | --- | --- | --- | --- | --- |
| **Continuous platform risk score** | 0.646 | 0.567–0.725 | NA^[3]^ | NA | NA |
| **Deployed platform risk category** | 0.512 | 0.477–0.539 | NA | NA | NA |
| **Enhanced behavioral model** | 0.728 | 0.656–0.801 | 0.040 | 0.688 | 1000 / 2623 |
| **Fully adjusted model** | 0.752 | 0.686–0.817 | 0.039 | 0.713 | 1000 / 2944 |

**Table S2. Pairwise DeLong comparisons of apparent model discrimination among MSM, Easy Test Know online HIVST platform, China, November 2023-February 2025**

| **Comparison** | **AUC** | | ***P* value** |
| --- | --- | --- | --- |
|  | **model 1** | **model 2** |  |
| Platform risk score vs Enhanced behavioral model | 0.646 | 0.728 | 0.0099 |
| Platform risk score vs Fully adjusted Firth model | 0.646 | 0.752 | <0.001 |

**Table S3. Apparent and bootstrap optimism-corrected calibration of reconstructed behavioral models**

| **Model** | **Estimate** | **Calibration-in-the-large** | **Calibration slope** | **E/O^[4]^ ratio** | **Brier score** | **HL^[5]^ χ² (df)** | ***P* value** |
| --- | --- | --- | --- | --- | --- | --- | --- |
| **Enhanced behavioral model** | Apparent | -0.149 | 1.031 | 1.144 | 0.005102 | 7.007 (7) | .428 |
| **Enhanced behavioral model** | Optimism-corrected | -0.141 | 0.940 | 1.137 | 0.005273 | NA | NA |
| **Fully adjusted Firth model** | Apparent | -0.162 | 1.035 | 1.158 | 0.005103 | 7.121 (8) | .524 |
| **Fully adjusted Firth model** | Optimism-corrected | -0.152 | 0.936 | 1.149 | 0.005292 | NA | NA |

**Table S4. High-risk venue diversity scores and observed HIV positivity among MSM, Easy Test Know online HIVST platform, China, November 2023-February 2025**

| **High-risk venue diversity score** | **HIV-negative, n (%)** | **HIV-positive, n (%)** |
| --- | --- | --- |
| **0** | **3 (100.0)** | **0 (0.0)** |
| **1** | **2742 (99.8)** | **6 (0.2)** |
| **2** | **3806 (99.5)** | **18 (0.5)** |
| **3** | **2741 (99.2)** | **21 (0.8)** |
| **4** | **610 (97.9)** | **13 (2.1)** |
| **5** | **1 (100.0)** | **0 (0.0)** |
| **6** | **0 (–)** | **0 (–)** |

Note: The linear trend was assessed using Firth logistic regression, with the high-risk venue diversity score treated as an ordinal continuous variable. Each one-category increase in the diversity score was associated with increased HIV positivity (OR=2.04, 95% CI: 1.53–2.74; P for trend <0.001).

**Table S5. Enhanced behavioral Firth logistic regression model** **for observed HIV positivity among 9,961 medium- and high-risk MSM, Easy Test Know online HIV self-testing platform, China, November 2023-February 2025**

| **Variables** | | **β coefficient** | **OR (95% CI)** | ***P* value** |
| --- | --- | --- | --- | --- |
| **Intercept** | |  |  |  |
|  | Constant | 1.972304 | — | — |
| **Age** | |  |  |  |
|  | <20 | 0.000000 | Reference | — |
|  | 20–29 | 1.324392 | 3.76 (0.50–481.42) | 0.253 |
|  | 30–39 | 0.724218 | 2.06 (0.29–262.07) | 0.567 |
|  | 40–49 | 1.385374 | 4.00 (0.49–518.04) | 0.241 |
|  | 50–59 | 0.380473 | 1.46 (0.004–307.01) | 0.867 |
|  | ≥60 | 0.835410 | 2.31 (0.01–428.30) | 0.681 |
| **Sexual_orientation** | |  |  |  |
|  | Uncertain | 0.000000 | Reference | — |
|  | Bisexual | -5.444086 | 0.004 (0.000–1.40) | 0.061 |
|  | Gay | -4.638378 | 0.010 (0.000–1.73) | 0.069 |
| **High_risk_venue_cat** | |  |  |  |
|  | 0 types | 0.000000 | Reference | — |
|  | 1 type | -3.958639 | 0.02 (0.001–5.22) | 0.133 |
|  | ≥2 types | -3.021722 | 0.05 (0.002–12.84) | 0.228 |
| **Condom_use** | |  |  |  |
|  | Consistent use | 0.000000 | Reference | — |
|  | Occasional use | 3.675861 | 39.48 (1.20–14,297.97) | 0.037 |
|  | No use | 4.210077 | 67.36 (1.90–25,053.06) | 0.016 |
| **HIV_positive_partner** | |  |  |  |
|  | Uncertain | 0.000000 | Reference | — |
|  | No | 1.894475 | 6.65 (2.91–13.70) | <0.001 |
|  | Yes | 5.232647 | 187.29 (8.45–29,386.56) | 0.002 |
| **Num_partners** | |  |  |  |
|  | >10 | 0.000000 | Reference | — |
|  | 2–5 | -4.828968 | 0.008 (0.002–0.04) | <0.001 |
|  | 6–9 | -4.410449 | 0.01 (0.002–0.07) | <0.001 |
| **Commercial_sex** | |  |  |  |
|  | No | 0.000000 | Reference | — |
|  | Yes | -0.232197 | 0.79 (0.11–3.42) | 0.777 |
| **STI_history** | |  |  |  |
|  | No | 0.000000 | Reference | — |
|  | Yes | 4.718290 | 111.98 (8.40–15,442.74) | <0.001 |
| **New_psychoactive_drug_use** | |  |  |  |
|  | No | 0.000000 | Reference | — |
|  | Yes | 0.149672 | 1.16 (0.55–2.80) | 0.711 |
| **Sexual_role** | |  |  |  |
|  | Both insertive and receptive | 0.000000 | Reference | — |
|  | Receptive | 0.082816 | 1.09 (0.49–2.81) | 0.850 |
|  | Insertive | -0.249007 | 0.78 (0.33–2.07) | 0.593 |
| **Group_sex** | |  |  |  |
|  | Uncertain | 0.000000 | Reference | — |
|  | No | 0.529076 | 1.70 (0.24–215.20) | 0.686 |
|  | Occasional | 1.108838 | 3.03 (0.34–399.25) | 0.383 |

1. AUC indicates area under the receiver operating characteristic curve.
2. Stratified bootstrap resampling (2000 replicates) estimated the 95% CIs for apparent AUCs.
3. NA indicates not applicable.
4. expected-to-observed ratio.
5. Hosmer-Lemeshow (HL) statistics are reported only for apparent calibration and were not optimism-corrected.
